# Sphingosine‐1‐Phosphate Receptor 2 Agonist Mobilises Endogenous Muse Cells to Repair Damaged Myocardial Tissue in Male Rabbits

**DOI:** 10.1111/jcmm.70447

**Published:** 2025-04-17

**Authors:** Shingo Minatoguchi, Yoshihisa Yamada, Noriko Endo, Hiromitsu Kanamori, Atsushi Mikami, Hiroyuki Okura, Shinya Minatoguchi

**Affiliations:** ^1^ Department of Cardiology Gifu University Graduate School of Medicine Gifu Japan; ^2^ Heart Failure Center Gifu Municipal Hospital Gifu Japan

**Keywords:** cardiac function, endogenous muse cells, infarct size, mobilisation, myocardial infarction, sphingosine‐1‐phosphate receptor 2 agonist

## Abstract

Muse cells, pluripotent stem cells present mainly in the bone marrow (BM) selectively accumulate to damaged tissue by sensing sphingosine‐1‐phosphate (S1P) and replace damaged cells by differentiating in situ. Acute myocardial infarction (AMI) model rabbits were subcutaneously injected either with Vehicle (*n* = 15), S1PR2‐agonist (*n* = 16), or S1PR2‐agonist + S1PR2‐antagonist (*n* = 10). The number of Muse cells in the peripheral blood was assessed by flow cytometry at 12 h after AMI. The S1PR2‐agonist group showed a significant increase in the peripheral‐blood Muse cell number at 12 h (*p* < 0.05), as well as infarct size reduction (*p* < 0.05) and improvement of left ventricular (LV) function (*p* < 0.05) at 2 weeks compared with the other 2 groups. The number of peripheral‐blood Muse cells positively correlated with LV ejection fraction (*p* < 0.05) and inversely correlated with infarct size (*p* < 0.05). Transplanted autologous green fluorescent protein (GFP)‐labelled BM‐Muse cells into the BM, followed by the administration of either Vehicle (*n* = 5) or S1PR2 agonist (*n* = 5) revealed a higher number of homed GFP‐Muse cells expressing the cardiac markers troponin‐I, α‐actinin, connexin‐43 and the vascular marker CD31 in the border areas in the S1PR2‐agonist group compared with the vehicle group. The mobilisation of endogenous Muse cells using S1PR2‐agonist may be a promising therapeutic approach.

## 1 Introduction

Acute myocardial infarction (AMI), particularly large and transmural infarctions, alters both infarcted and non‐infarcted regions of the left ventricle (LV), resulting in a thinner LV wall and greater LV dilation, termed LV remodelling, and leading to a deterioration of LV function and a poor prognosis for survival [1]. Despite extensive efforts to prevent AMI and improve outcomes, AMI remains a leading cause of morbidity and mortality worldwide [2, 3]. Even when coronary reperfusion is successful, deterioration of the LV ejection fraction (LVEF) and LV remodelling often occur after AMI and these factors predict the long‐term prognosis; higher recovery of the LVEF and smaller LV dilation are associated with a better long‐term prognosis and the converse is also true [4, 5]. Therefore, fundamental treatment following AMI is critically required to repair the infarcted myocardial tissues, improve the LV function, attenuate LV dilation and improve the prognosis.

Multi‐lineage differentiating stress‐enduring (Muse) cells are non‐tumorigenic pluripotent‐like stem cells identified as pluripotent surface marker stage‐specific embryonic antigen‐3 positive (SSEA‐3+) cells in the bone marrow (BM; corresponding to ~0.03% of the mononucleated fraction), peripheral blood (~0.04% of mononucleated cells) and connective tissue of various organs [6, 7, 8, 9, 10]. Muse cells are unique because: (1) the number of endogenous Muse cells in the peripheral blood, possibly mobilised from the BM, rapidly increases after tissue damage in stroke and myocardial infarction patients [8, 11], as well as after cardiac rehabilitation, which improves the prognosis of AMI [12] and (2) intravenously administered exogenous Muse cells selectively home to the damaged tissue and replace damaged/apoptotic cells by spontaneous differentiation into the appropriate cell type for tissue repair [13, 14, 15, 16, 17, 18]. We recently reported that AMI patients with higher mobilisation of endogenous peripheral‐blood Muse cells in the acute phase exhibit statistically meaningful improvement of LV function and attenuation of LV remodelling in the chronic phase at 6 months after onset when compared with patients who exhibit no such increase in the mobilisation of peripheral‐blood Muse cells in the acute phase [8]. This observation suggested that the number of endogenous peripheral‐blood Muse cells correlates with reparative activity. In animal models of AMI [13], stroke, epidermolysis bullosa, chronic kidney disease, liver cirrhosis and corneal scarring [10, 14, 15, 16, 17], intravenous administration of exogenous Muse cells leads to efficient tissue repair and functional recovery. In a rabbit AMI model, we recently reported that specific homing of intravenously infused exogenous Muse cells into the infarcted myocardium is mediated through the sphingosine‐1‐phosphate (S1P)‐S1P receptor 2 (S1PR2) axis signalling pathway—an interaction between S1P produced by the damaged heart and S1PR2 expressed in Muse cells [13]. After selective homing, Muse cells spontaneously differentiate into physiologically functional cardiomyocytes and blood vessels within the damaged tissue, resulting in a significantly reduced infarct size, improved LV function and attenuated LV remodelling [13]. Moreover, allogeneic Muse cells are able to escape host immunorejection after intravenous administration and survive in the host tissue as differentiated cells for over 6 months, even without immunosuppressive treatment, which is partly explained by the Muse cell expression of human leukocyte antigen (HLA)‐G, which mediates immune tolerance in the placenta [13]. On the basis of these highly unique properties, intravenously administered donor‐derived Muse cells have been applied to clinical trials for the treatment of AMI [19], epidermolysis bullosa [20], ischaemic stroke [21] and amyotrophic lateral sclerosis [22] under the approval of regulatory authorities, all without HLA matching or treatment with long‐term immunosuppressants. These results suggested that the utilisation of Muse cells may provide a novel strategy for clinical treatment [23].

We considered that inducing the efficient mobilisation of endogenous‐Muse cells from the BM into the peripheral circulating blood could promote Muse cell homing to the damaged post‐infarct tissue and repair the tissue similarly to exogenously administered Muse cells. Activation of endogenous Muse cells might create less of a burden on the patient, and improve patient treatment costs and accessibility to the strategy of using expanded autologous and donor‐derived Muse cells. While S1P is a candidate molecule for inducing the mobilisation of endogenous Muse cells, it could also evoke the mobilisation of inflammatory cells through S1PR1 [24]. We therefore hypothesised that an S1PR2 agonist, rather than S1P, could be an efficient selective mobiliser of endogenous Muse cells. The purpose of this study was to investigate whether post‐infarct treatment with an S1PR2 agonist efficiently mobilises endogenous Muse cells into the peripheral blood, and whether the mobilised Muse cells engraft into the post‐infarct tissue, differentiate into cardiac and vessel cells and deliver statistically meaningful tissue repair and functional recovery in a rabbit model of AMI.

## 2 Materials and Methods

In this study, all rabbits received humane care, and the experiments were carried out in strict accordance with the recommendations of the Standards Relating to the Care and Management of Laboratory Animals and Relief of Pain (2006) published by the Japanese Ministry of the Environment and with the Guide for the Care and Use of Laboratory Animals, published by the US National Institutes of Health (NIH Publication, 8th Edition, 2011). The study protocol was approved by the Committee for Animal Research and Welfare of Gifu University, Gifu, Japan (Permit Number: 25–10). The experiment was performed according to the ARRIVE Guidelines(https://www.nc3rs.org.uk/arrive‐guidelines).

### 2.1 Brief Outline of the Protocols of the In Vitro and In Vivo Experiments

#### 2.1.1 In Vitro Experiments

##### 2.1.1.1 In Vitro Muse Cell Migration Assessment

Using a Matrigel invasion chamber, mobilisation of Muse cells was examined in the presence or absence of an S1PR2‐specific antagonist (JTE‐013) and an S1PR2 agonist (SID46371153). Details are provided in the Supporting Information.

##### 2.1.1.2 In Vitro Differentiation of Rabbit Peripheral‐Blood Muse Cells Into Cardiac‐Lineage Cells

Peripheral blood which contained Muse cells was obtained from the rabbits at 2 h after injecting SID46371153. Cardiac‐lineage differentiation was analysed. The collected SSEA‐3^+^ cells were subjected to suspension culture and the differentiation of blood Muse cells into cardiac‐lineage cells was evaluated. Details are provided in the Supporting Information.

##### 2.1.1.3 Reverse Transcription‐Quantitative Polymerase Chain Reaction

Rabbit cardiac troponin T and α‐actinin mRNAs were quantified using the One Step SYBR PrimeScript RT‐PCR Kit and the Thermal Cycler Dice Real Time System II. Details are provided in the Supporting Information.

##### 2.1.1.4 Immunocytochemistry

Cardiac troponin T and sarcomeric α‐actinin were stained. Details are provided in the Supporting Information.

#### 2.1.2 In Vivo Experiments

##### 2.1.2.1 Rabbit AMI Model

Rabbit AMI model was made according to the method previously reported [13, 25, 26].

Details are provided in the Supporting Information.

##### 2.1.2.2 Injection of an S1PR2 Agonist and Antagonist Into the AMI Rabbits

Thirty minutes after reperfusion, the rabbits received a subcutaneous injection of either DMSO (vehicle group), 10 mg/kg SID46371153 (S1PR2 agonist group), or 5 mg/kg JTE‐013 + 10 mg/kg SID46371153 (S1PR2 agonist + antagonist group). Details are provided in the Supporting Information.

##### 2.1.2.3 Flow Cytometric Analysis

The number of circulating Muse cells in the peripheral blood was measured as cells double‐positive for SSEA‐3^+^, a marker of pluripotency and CD44^+^, a marker of mesenchymal stem cells, at 12 h after AMI. Details are provided in the Supporting Information.

##### 2.1.2.4 Plasma Troponin T Measurement

Plasma troponin T levels were measured at 12 h after AMI in rabbits.

##### 2.1.2.5 Physiologic Studies

On day 14 after AMI, blood pressure, heart rate, LVEF, LVFS, LVDd, LVDs, peak +dP/dt, peak −dP/dt were measured by echocardiography and via a catheter. Echocardiography (SSD2000, Aloka Co. Ltd., Tokyo, Japan) was performed, and the LVEF, LV fractional shortening (LVFS) and LV end‐diastolic (LVDd) and end‐systolic dimensions (LVDs) were obtained at 14 days after AMI. We measured these parameters because long‐term prognosis of AMI is determined by LVEF and LV remodelling [4, 5]. Details are provided in the Supporting Information.

##### 2.1.2.6 Myocardial Infarct Size Measurement

Myocardial infarct size was measured at 14 days after AMI. Details are provided in the Supporting Information.

##### 2.1.2.7 CD31‐Positive Microvessels and Cardiomyocyte Apoptosis

CD31‐positive microvessels and cardiomyocyte apoptosis were measured 14 days after AMI. Details are provided in the Supporting Information.

##### 2.1.2.8 Transplantation of Autologous Green Fluorescent Protein‐Labelled Muse Cells Into the BM Followed by Administration of an S1PR2 Agonist

BM aspirate was collected from the left or right iliac crest of each rabbit, and mononuclear cells were isolated. GFP‐labelled Muse cells were obtained from GFP‐labelled MSCs by incubating them with an anti‐SSEA‐3 antibody. Autologous BM‐Muse cells labelled with GFP (approximately 3 × 10^5^ cells) were returned to the BM cavity of the left and right iliac crests in each rabbit. At 48 h later, rabbits underwent 30 min of coronary occlusion and reperfusion, and then, 0.5 mL DMSO (*n* = 5) or 10 mg/kg SID46371153 (S1PR2 agonist, *n* = 5) was subcutaneously administered 30 min after coronary reperfusion, and the rabbits were followed up for 2 weeks. Details are provided in the Supporting Information.

##### 2.1.2.9 Immunohistochemistry Using Cryosections

Two weeks after AMI, rabbits were sacrificed and immunohistochemistry using cryosections of the heart, GFP, troponin I, connexin 43 and CD31 was stained. Details are provided in the Supporting Information.

##### 2.1.2.10 Measurement of Plasma Levels of SID46371153 (S1PR2 Agonist)

Blood samples were collected from the ear artery at 12 h after AMI to measure plasma levels of the S1PR2 agonist SID46371153 in each group. Details are provided in the Supporting Information.

### 2.2 Statistical Analysis

All values are presented as the mean ± standard error (SE). The normality of the data distributions was tested using the Kolmogorov–Smirnov test. The significance of differences between 2 groups for normally distributed variables was determined by the unpaired Student's *t*‐test. The significance of differences amongst more than 3 groups for normally distributed variables was evaluated by 1‐way analysis of variance followed by multiple comparisons with the Tukey or Dunnett method. Correlation coefficients between 2 variables were obtained by linear regression analysis. All statistical analyses were performed using GraphPad Prism 7 (GraphPad Software Inc.). Values of *p* < 0.05 (*) were considered significant, and values of *p* < 0.01 (**) and *p* < 0.001 (***) were considered highly significant.

## 3 Results

### 3.1 Effect of S1PR2 Agonist and Antagonist on Rabbit Muse Cell Migration In Vitro

**FIGURE 1:**
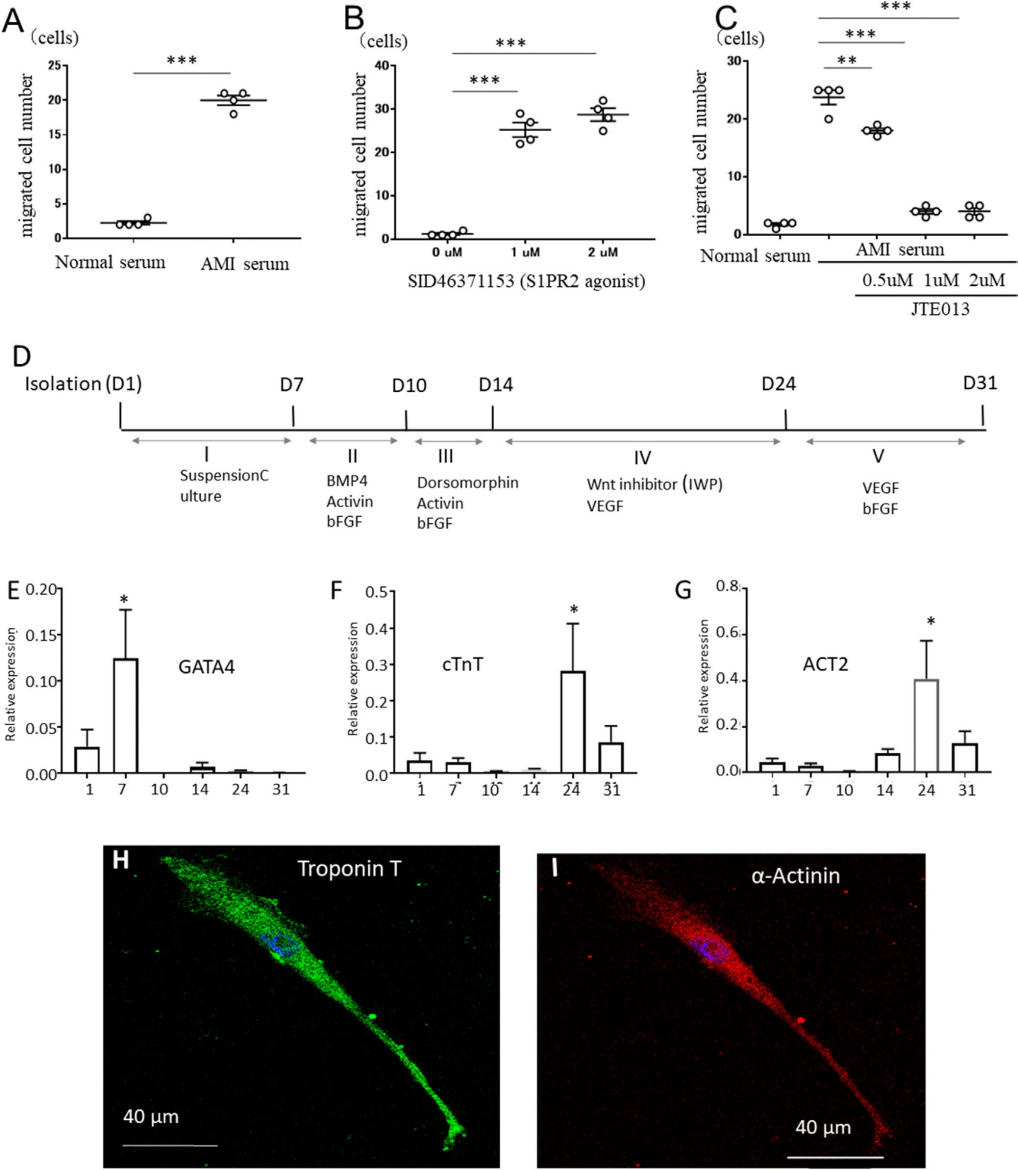
In vitro analysis of Muse cell migration and cardiac differentiation. (A–C) Migration assay of Muse cells. Migration of rabbit Muse cells toward rabbit AMI serum (A), S1PR2 agonist SID46371153 (B). Suppression of rabbit Muse cell migration toward rabbit AMI serum by the S1PR2 antagonist JTE‐013 (C). (D–I) Cardiac differentiation of rabbit peripheral‐blood Muse cells. (D) Protocol of cardiac differentiation. Stage I, suspension culture; Stage II, mesodermal induction; Stage III, BMP4 inhibition; Stage IV, cardiac‐endothelial progenitor induction by Wnt product inhibition; Stage V, cardiac‐lineage stabilisation. Reverse transcription‐quantitative polymerase chain reaction of GATA4 (E), cardiac troponin T (cTnT) (F) and sarcomeric α‐actinin (ACT2) from the Stage I to Stage V is shown. Expression of GATA4 was maximum at day 7, and cTnT and ACT2 were maximum at day 24 with statistical significance compared to day 1 (*p* < 0.05). (H and I): Immunostaining of rabbit peripheral‐blood Muse cells after cardiac differentiation (Stage V) expressing troponin T (H) and α‐actinin (I). (A) *N* = 5 in each bar, *p* < 0.001, unpaired Student's *t*‐test, (B and C) *N* = 5 in each bar, *p* < 0.01 and *p* < 0.001, 1‐way analysis of variance followed by multiple comparisons with the Dunnett method, (E–G) *N* = 5 in each bar of day 1, 7, 10, 17, 24 and 31, *p* < 0.05, 1‐way analysis of variance followed by multiple comparisons with the Dunnett method, *p < 0.05, **p < 0.01, ***p < 0.001.

In the in vitro migration assay, a significantly greater number of rabbit BM‐Muse cells actively migrated to the rabbit AMI serum compared with the normal intact rabbit serum (*p* < 0.001; Figure 1A). In addition, Muse cells migrated toward the S1PR2 agonist SID46371153 in a concentration‐dependent manner (Figure 1B). On the other hand, the S1PR2 antagonist JTE‐013 efficiently suppressed the migration of Muse cells toward the AMI serum in a concentration‐dependent manner (Figure 1C). These findings indicate that the S1PR2 agonist and antagonist controlled the migration of rabbit peripheral‐blood Muse cells.

### 3.2 Cardiac Differentiation Potential of Peripheral‐Blood Muse Cells

To validate whether endogenous Muse cells in the rabbit peripheral blood have the potential to differentiate into cardiac‐lineage cells, Muse cells were subjected to cardiomyocyte induction according to a previous report [27] (Figure 1D). Muse cells collected from the rabbit peripheral blood were directly cultured in suspension for 7 days (Stage I); treated with BMP‐4, activin and bFGF for 3 days (mesodermal induction, Stage II); followed by dorsomorphin, activin and bFGF for 4 days (BMP4 inhibition, Stage III); inhibitor of WNT production‐3 and VEGF for 10 days (cardiac‐endothelial progenitor induction, Stage IV), and finally with VEGF and bFGF for 7 days (cardiac‐lineage stabilisation, Stage V).

Quantitative polymerase chain reaction revealed maximum expression of the early cardiac marker GATA4 at 7 days, the final stage of suspension culture (Figure 1E). Expression of the late markers cardiac troponin T (Figure 1F) and sarcomeric α‐actinin (ACT2; Figure 1G) was maximum at day 24, the end of the cardiac –endothelial progenitor induction stage (Stage IV in Figure 1D). Immunocytochemistry demonstrated that rabbit peripheral‐blood Muse cells expressed cardiac troponin T (Figure 1H) and sarcomeric α‐actinin (Figure 1I). Negative control cells incubated only with the secondary antibody did not show any signal (data not shown). Together, these data suggest that rabbit peripheral‐blood Muse cells have the potential to differentiate into cardiac‐lineage cells.

### 3.3 Mobilisation of Muse Cells Into the Peripheral Blood Promoted by an S1PR2 Agonist

AMI rabbits received a subcutaneous injection of DMSO (Vehicle group) or S1PR2 agonist (S1PR2 agonist group) at 30 min after coronary reperfusion, and the peripheral blood was analysed by flow cytometry 12 h after AMI. On the basis of flow cytometric analysis, human Muse cells are contained mainly in the mononuclear cell fraction of the peripheral blood [8]; therefore, we gated the mononuclear area comprising the monocyte and lymphocyte fractions in the rabbit peripheral blood.

**FIGURE 2:**
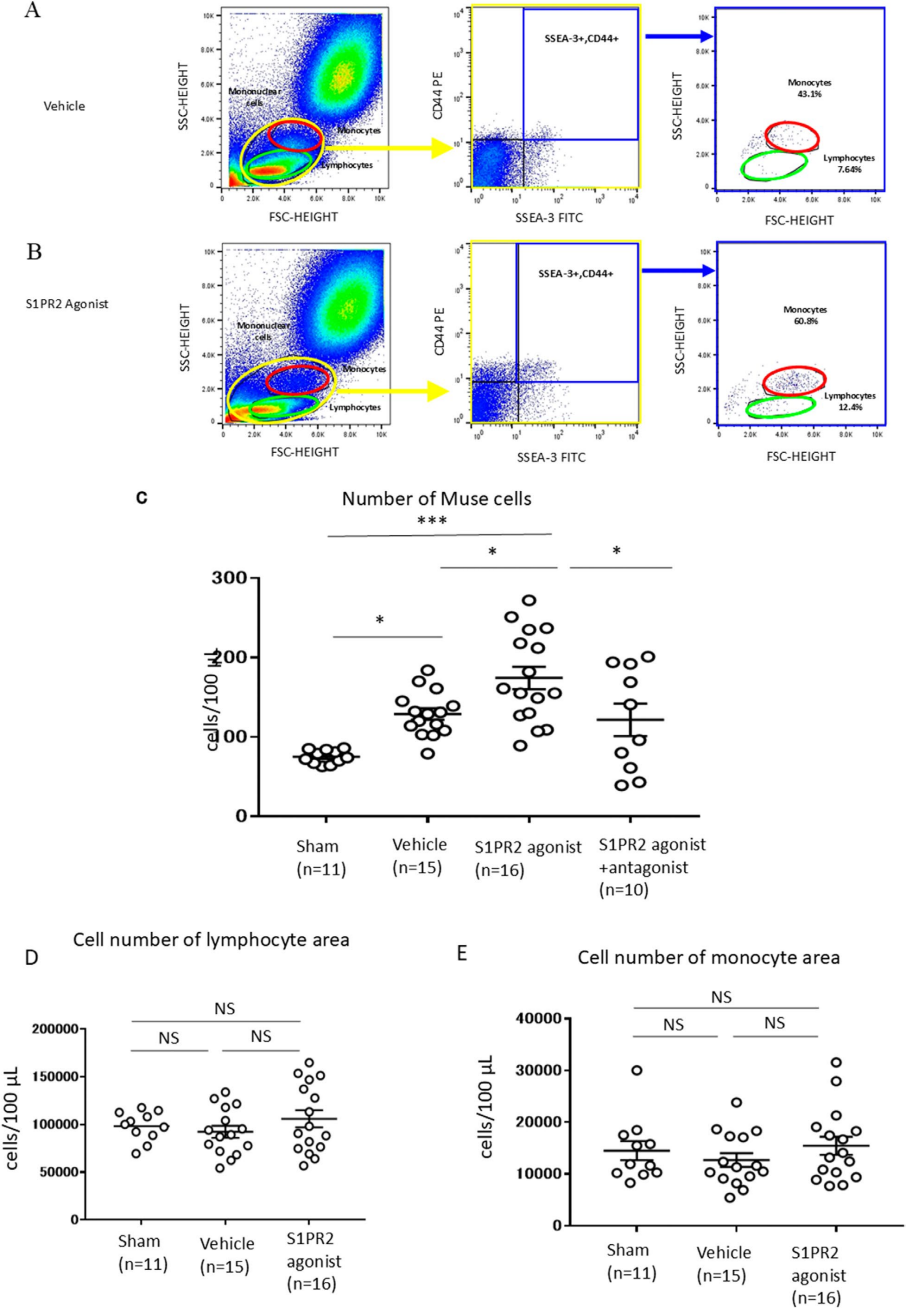
Flow cytometry of the rabbit peripheral blood after S1PR2 agonist treatment. (A and B) Typical images of flow cytometry in the Vehicle (A) and S1PR2 agonist (B) groups. We gated the mononuclear cell area, and monocyte and lymphocyte areas on the basis of dot plot, one of the display types of FlowJo software (forward scatter [FSC] and side scatter [SSC] of the flow cytometer were focused on mononuclear cells; all cells). Distribution of SSEA‐3/CD44 double‐positive Muse cells in the mononuclear cell area, monocyte area, and lymphocyte area within the gated area (Muse cells). Percent of Muse cells in the monocyte and lymphocyte areas is indicated as the percent of Muse cells to that in the total mononuclear cell area. Number of Muse cells in the monocyte area was defined as the number of Muse cells in the peripheral blood because the majority of Muse cells were detected in the monocyte area and fewer Muse cells were detected in the lymphocyte area. (C) Number of SSEA‐3/CD44‐double‐positive Muse cells in the peripheral blood of the sham (*n* = 11), Vehicle (*n* = 15), S1PR2 agonist (*n* = 16), and S1PR2 agonist + antagonist (*n* = 10) groups. *p < 0.05, ***p < 0.001, 1‐way analysis of variance followed by multiple comparisons with the Tukey method. (D) Number of whole cells in the lymphocyte area in the sham (*n* = 11), Vehicle (*n* = 15), and S1PR2 agonist (*n* = 16) groups. No difference was detected amongst the 3 groups. 1‐way analysis of variance followed by multiple comparisons with the Tukey method. (E) Number of whole cells in the monocyte area in the sham (*n* = 11), Vehicle (*n* = 15), and S1PR2 agonist (*n* = 16) groups. No difference was detected amongst the 3 groups. 1‐way analysis of variance followed by multiple comparisons with the Tukey method, NS; not significant.

Typical flow cytometry results in the Vehicle and S1PR2 agonist groups are shown in Figure 2A,B, respectively. The results demonstrated that Muse cells were mainly included in the monocyte area and fewer Muse cells were included in the lymphocyte area in both the vehicle and S1PR2 agonist groups; in the Vehicle group, ~43.1% of Muse cells were in the monocyte area and ~ 7.6% were in the lymphocyte area, and in the S1PR2 agonist group, ~60.8% were in the monocyte area and ~ 12.4% were in the lymphocyte area (Figure 2A,B). Therefore, Muse cell measurement was performed by focusing on the monocyte region.

The number of SSEA‐3^+^/CD44^+^‐Muse cells was the greatest in the S1PR2 agonist group (174 ± 14/100 μL) compared with the sham (*p* < 0.0001), Vehicle (*p* < 0.05), and S1PR2 agonist +antagonist (JTE013) (*p* < 0.05) groups at 12 h after AMI (Figure 2C). A higher number of Muse cells was detected in the Vehicle group (130 ± 7/100 μL) compared with the sham group (75 ± 3/100 μL, *p* < 0.05; Figure 2C). The increased number of Muse cells was abolished by co‐injection of JTE013 in the S1PR2 agonist + antagonist group (122 ± 20/100 μL), with the Muse cell number in the peripheral blood similar to that in the Vehicle group. The number of SSEA‐3^+^/CD44^+^‐Muse cells detected in the S1PR2 agonist group was significantly higher than that detected in the S1PR2 agonist + antagonist groups (*p* < 0.05; Figure 2C). Subcutaneously administered SID46371153 was confirmed to be transferred to the blood by measuring the plasma level of SID46371153 in all 4 groups; similar levels of SID46371153 were detected in the S1PR2 agonist and S1PR2 agonist + antagonist groups at 12 h after AMI, but not in the sham and Vehicle groups (Figure S1).

The number of whole cells in the lymphocyte area (Figure 2D) and in the monocyte area (Figure 2E) did not differ significantly amongst the sham, Vehicle and S1PR2 agonist groups, suggesting that the S1PR2 agonist did not evoke the mobilisation of immune cells into the peripheral blood at 12 h after administration.

### 3.4 Survival Rate of AMI Experimental Groups

To create a myocardial infarction model, 54 rabbits were initially enrolled. Amongst these animals, 10 were excluded because of technical problems, and 3 were excluded because of death during the 12 h after the operation. One rabbit died in the vehicle group, one rabbit died in the S1PR2 agonist group, and one rabbit died in the S1PR2 agonist + antagonist group. Thus, the experiments were completed in the remaining 41 rabbits, and the data from these animals were used for the analysis.

### 3.5 Physiologic Findings

**FIGURE 3:**
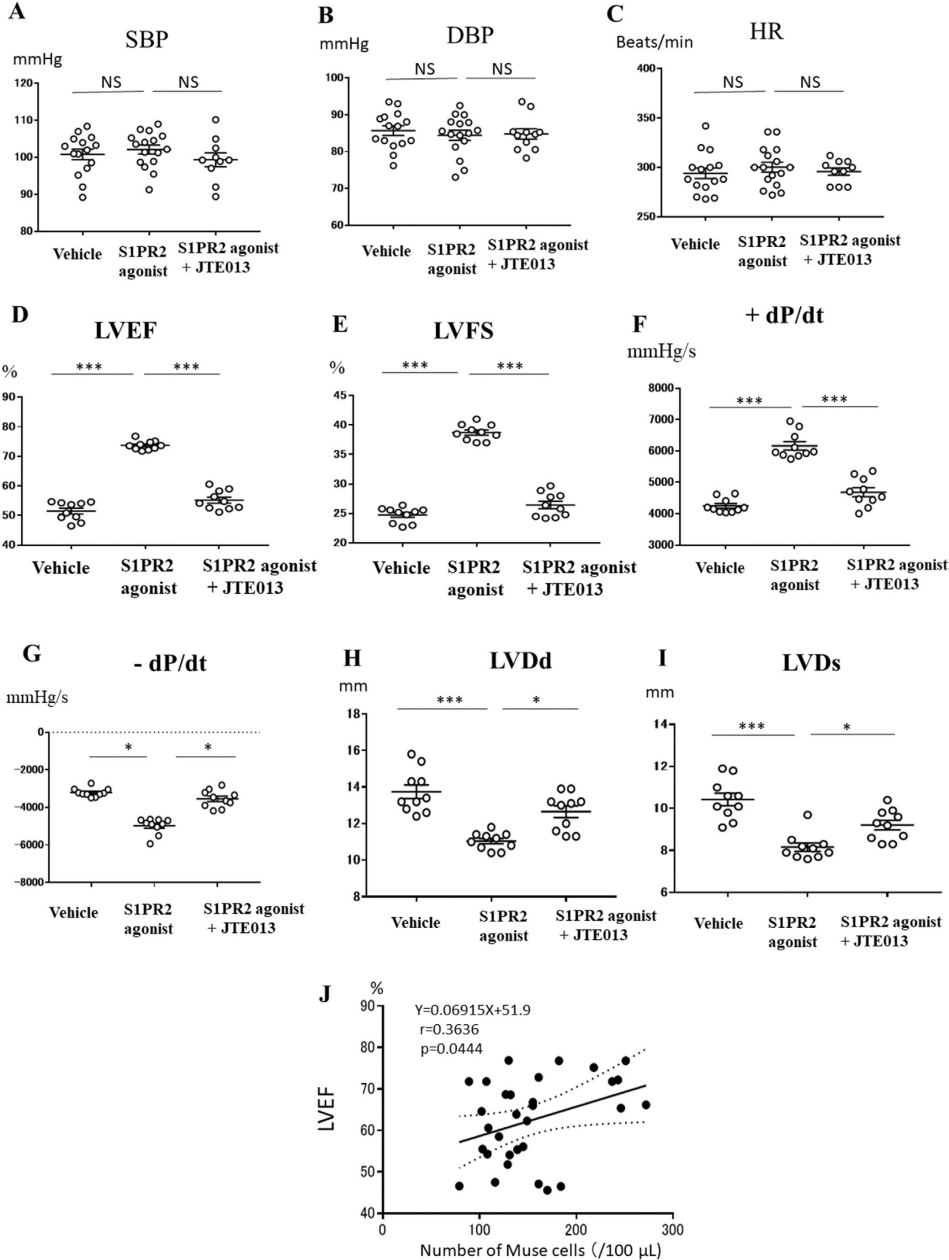
Physiologic analysis. Systolic blood pressure (SBP) (A), diastolic blood pressure (DBP) (B), heart rate (HR) (C), LV ejection fraction (LVEF) (D), LV fractional shortening (LVFS) (E), LV end‐diastolic diameter (LVDd) (H), and LV end‐systolic diameter (LVDs) (I) were assessed by echocardiography in the Vehicle, S1PR2 agonist, and S1PR2 agonist + antagonist groups. The peak +dP/dt (F) and peak −dP/dt (G) were assessed using a Millar catheter. (J) Relationship between the number of Muse cells in the peripheral blood after AMI and LV function. (A–I) Vehicle (*n* = 15), S1PR2 agonist group (*n* = 16), S1PR2 agonist + antagonist group (*n* = 10), *p < 0.05, ***p < 0.001: 1‐way analysis of variance followed by multiple comparisons with the Tukey method, (J) *N* = 31 (Vehicle and S1PR2 agonist groups), *p* < 0.05, linear regression analysis.

We evaluated the cardiac function in rabbits receiving Vehicle, S1PR2 agonist and S1PR2 agonist + antagonist at 2 weeks after AMI. No differences in the systolic and diastolic blood pressures or heart rate were detected amongst the Vehicle, S1PR2 agonist and S1PR2 agonist + antagonist groups (Figure 3A–C). In echocardiography and cardiac catheterisation studies, however, the LVEF, LVFS, +dP/dt and −dP/dt values were significantly higher in the S1PR2 agonist group compared with the Vehicle and S1PR2 agonist + antagonist groups (LVEF: *p* < 0.001 compared with the Vehicle and S1PR2 agonist + antagonist groups [Figure 3D], LVFS: *p* < 0.001 compared with the Vehicle and S1PR2 agonist + antagonist groups [Figure 3E], peak +dP/dt: *p* < 0.001 compared with the Vehicle and S1PR2 agonist + antagonist groups [Figure 3F], and peak −dP/dt: *p* < 0.05 compared with the Vehicle and S1PR2 agonist + antagonist groups [Figure 3G]), but the LVDd and LVDs values were significantly lower (LVDd: *p* < 0.001 compared with the Vehicle group and *p* < 0.05 compared with the S1PR2 agonist + antagonist group [Figure 3H] and LVDs: *p* < 0.001 compared with the Vehicle group and *p* < 0.05 compared with the S1PR2 agonist + antagonist group [Figure 3I]). The number of Muse cells in the peripheral blood positively correlated with the LVEF values (*p* < 0.05) (Figure 3J). The LV end‐diastolic wall thickness and LV end‐systolic wall thickness at the infarct border area and remote area were shown in Figure S3.

### 3.6 Myocardial Infarct Size

**FIGURE 4:**
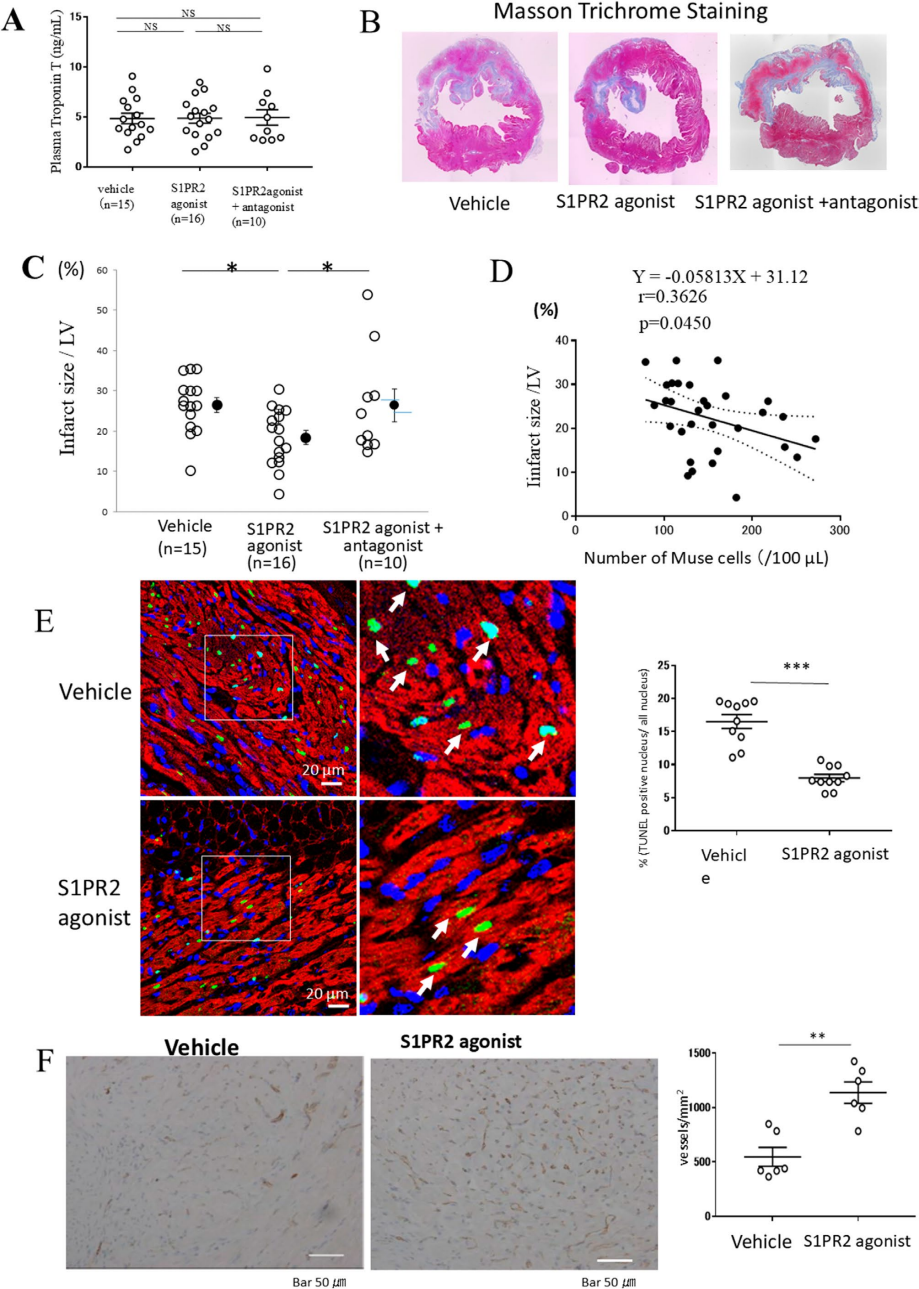
Myocardial infarct size and histologic analysis. (A) Plasma troponin T levels at 12 h after AMI in Vehicle, S1PR2 agonist and S1PR2 agonist + antagonist groups. (B) Typical figures of transverse LV sections at the papillary muscle level stained by Masson‐trichrome at 2 weeks. (C) The infarct size as a percent of the total LV area amongst the 3 groups at 2 weeks. Vehicle (*n* = 15), S1PR2 agonist group (*n* = 16), S1PR2 agonist + antagonist group (*n* = 10), *p* < 0.05, 1‐way analysis of variance followed by multiple comparisons with the Tukey method. (D) Relation between the number of Muse cells in the peripheral blood after AMI and the infarct size. *n* = 31 (Vehicle and S1PR2 agonist groups), *p* < 0.05, linear regression analysis. (E) TUNEL‐positive cardiomyocytes in the Vehicle (*n* = 5) and S1PR2 agonist (*n* = 5) groups at 3 days. Red signal: Cardiomyocytes stained by myoglobin, Green signal: Apoptotic cells detected by TUNEL, Blue signal: Counterstain by Hoechst 33342. 20 observations in each, *p* < 0.001, unpaired Student's *t*‐test. (F) CD31‐positive cells at the border area in the Vehicle (*n* = 5) and S1PR2 agonist (*n* = 5) groups at 2 weeks. 20 observations in each, *p* < 0.01, unpaired Student's *t*‐test. *p < 0.05, **p < 0.01, ***p < 0.001.

Plasma troponin T levels, which closely correlate with infarct size [28], were substantially elevated at 12 h after AMI in all three groups with no significant differences amongst groups (Figure 4A), suggesting equivalent infarction induction. Figure 4B shows typical examples of a transverse LV section at the papillary muscle level stained by Masson trichrome at 2 weeks after AMI. The infarct size shown as the percentage of the total LV area was significantly smaller in the S1PR2 agonist group (18.4% ± 1.8%) than in the Vehicle group (26.5% ± 1.8%, *p* < 0.05; Figure 4C). The reduction of the infarct size by the S1PR2 agonist was abolished by the presence of JTE013, a S1PR2 antagonist (26.4% ± 4.1%, *p* < 0.05; Figure 4B,C). The number of Muse cells in the peripheral blood inversely correlated with the infarct size (*r* = 0.3626, *p* < 0.05; Figure 4D).

The myocardial infarct size as a percentage of the area at risk was significantly smaller in the S1PR2 agonist group (14.2% ± 1.9%, *n* = 8) than in the Vehicle group (32.1% ± 5.5%, *n* = 8, *p* < 0.001) (Figure S4). The inflammatory cells were observed at the infarct border area both in the Vehicle and S1PR2 agonist groups, but the number of inflammatory cells were not different between both groups at 2 weeks after AMI. The inflammatory cells were hardly observed in the remote area both in the Vehicle and S1PR2 agonist groups (Figure S5).

### 3.7 Effect of the S1PR2 Agonist on Cardiomyocyte Apoptosis

On day 3 after AMI, TUNEL‐positive myocytes were observed in the peri‐infarct areas, and the number of TUNEL‐positive cardiomyocytes was significantly reduced in the S1PR2 agonist group (7.9% ± 0.5%) compared with the Vehicle group (16.5% ± 1.0%, *p* < 0.001; Figure 4E). Apoptotic cardiomyocytes were hardly observed in the remote area.

### 3.8 Effect of the S1PR2 Agonist on CD31‐Positive Microvessels

At 2 weeks after AMI, CD31^+^ (vascular endothelial cell marker) vessels were observed in the peri‐infarct areas. The density of the CD31^+^ microvessels (capillary density) was significantly greater in the infarct border area of the S1PR2 agonist group (1139 ± 98/mm^2^) than in the Vehicle group (547 ± 87/mm^2^, *p* < 0.01; Figure 4F). The number of CD31^+^ microvessels was small in the remote area in both the Vehicle and S1PR2 agonist groups and seemed to show no difference between the 2 groups.

### 3.9 Differentiation Marker Expression in Engrafted Muse Cells

To trace the engraftment of Muse cells mobilised by the S1PR2 agonist into the post‐infarct heart, rabbits underwent transplantation of autologous GFP‐labelled rabbit Muse cells into the BM, which is considered a Muse cell reserve [6, 8], and then S1PR2 agonist was injected subcutaneously 30 min after AMI.

**FIGURE 5:**
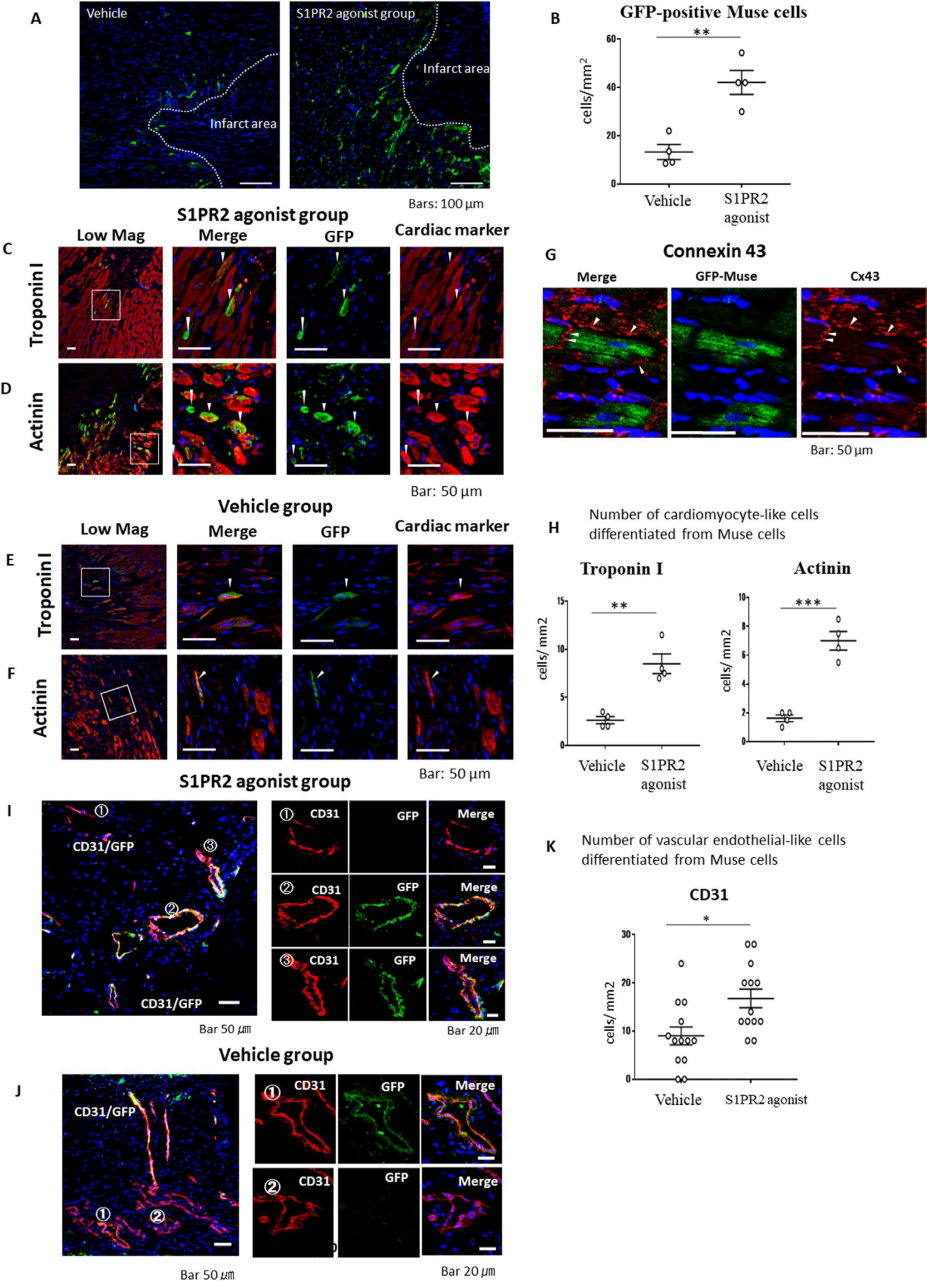
Cardiac marker expression in the vehicle and S1PR2 agonist groups at 14 days after AMI. (A) GFP‐positive Muse cells engrafted in the infarct and border areas of the myocardium in the Vehicle (*n* = 5) and S1PR2 agonist (*n* = 5) groups. (B) Number of GFP‐positive Muse cells engrafted in the infarct and border areas of the myocardium in the Vehicle and S1PR2 agonist groups (20 observations in each, *p* < 0.05, unpaired student's *t*‐test). (C–F) GFP‐positive Muse cells expressed troponin I and sarcomeric α‐actinin, markers of cardiomyocytes in the S1PR2 agonist (C and D) and Vehicle (E and F) groups. (G) GFP‐positive Muse cells expressed connexin 43, a marker of gap junction. (H) Number of GFP‐ and troponin I‐double‐positive cells, and GFP‐ and sarcomeric α‐actinin‐double‐positive cells in the Vehicle and S1PR2 agonist groups (20 observations in each, *p* < 0.05, unpaired student's *t*‐test). (I and J) GFP‐positive Muse cells expressed CD31, a marker of vascular endothelial cells, in the S1PR2 agonist (I) and Vehicle (J) groups. (K) Number of GFP‐ and CD31 double‐positive cells in the Vehicle and S1PR2 agonist groups. (20 observations in each, *p* < 0.05, unpaired student's *t*‐test) *p < 0.05, **p < 0.01, ***p < 0.001.

Two weeks later, GFP‐Muse cells were detected in the infarct border area of the myocardium, and the number of integrated Muse cells was significantly greater in the S1PR2 agonist group than in the Vehicle group (*p* < 0.01; Figure *5*A,B). No GFP‐labelled Muse cells were observed in the remote area. Engrafted GFP‐Muse cells expressed cardiac troponin‐I (a cardiomyocyte‐specific marker) and sarcomeric α‐actinin (a marker for actin‐binding protein that forms the Z line of cardiac myofibrils) in the infarct border area both in the S1PR2 agonist (Figure 5C,D) and control groups (Figure 5E,F). Connexin43 was detected between the host and GFP^+^ Muse cells in the S1PR2 agonist group (Figure 5G). The number of GFP^+^/cardiac troponin^+^ cells was significantly greater in the S1PR2 agonist group (8.5 ± 0.7 cells/mm^2^) than in the Vehicle group (2.4 ± 0.3 cells/mm^2^, *p* < 0.01; Figure 5H). The number of GFP^+^/α‐actinin I^+^ cells was significantly greater in the S1PR2 agonist group (6.4 ± 0.4/mm^2^) than in the Vehicle group (1.8 ± 0.0/mm^2^, *p* < 0.001; Figure 5H).

GFP‐Muse cells were also incorporated into vessels as CD31^+^ cells in the S1PR2 agonist and Vehicle groups (Figure 5I,J). The number of GFP and CD31 double‐positive cells was significantly greater in the S1PR2 agonist group (16.5 ± 2.1/mm^2^) than in the Vehicle group (9.0 ± 2.0/mm^2^, *p* < 0.05; Figure 5K).

## 4 Discussion

The present study demonstrated that post‐infarct treatment with an S1PR2 agonist compared with Vehicle in a rabbit model of AMI significantly: (1) increased mobilisation of endogenous SSEA‐3^+^/CD44^+^‐Muse cells in the peripheral blood; (2) increased the number of engrafted Muse cells in the infarct border area; (3) reduced the myocardial infarct size, improved LV function and attenuated LV remodelling, partly due to the differentiation of Muse cells into troponin I^+^, α‐actinin^+^, and connexin43^+^ cardiac‐lineage cells as well as into CD31^+^ vascular cells; (4) reduced the number of TUNEL‐positive cardiomyocytes in the peri‐infarct areas and (5) increased the vascular density at the infarct border area.

The number of SSEA‐3^+^/CD44^+^‐Muse cells in the peripheral blood increased after AMI (Vehicle group) compared with the baseline level (sham group), suggesting that AMI itself leads to the mobilisation of endogenous Muse cells into the peripheral blood. This finding is consistent with our recent report that endogenous Muse cells are mobilised into the peripheral blood in patients with AMI in the acute phase, and the number of mobilised Muse cells positively correlates with plasma S1P levels [8]. Furthermore, we have previously demonstrated that Muse cells but not non‐Muse cells migrated towards a S1PR2‐specific agonist (SID46371153) in a dose‐dependent manner, suggesting tight involvement with the S1P–S1PR2 axis in an in vitro study [13]. Although Muse cells without silencing S1PR2 migrated toward the S1PR2‐specific agonist SID46371153, Muse cells with silenced S1PR2 did not migrate towards S1D46371153 [13]. Furthermore, in a rabbit model of AMI, Muse cells migration toward the infarct area was blocked by the S1PR2‐specific antagonist JTE‐013 [13]. In addition, it has very recently been reported that endogenous Muse cells might be mobilised and accumulated to the myocardial tissues and might repair injured myocardium in patients with fulminant myocarditis [29]. The present our data further confirm previous experimental evidence of Muse cells‐mediated cardioprotection, and this is the first report demonstrating that S1PR2 agonist mobilises Muse cells into the peripheral blood and repair the damaged myocardium in a rabbit model of AMI.

Subcutaneously administered SID46371153 (S1PR2 agonist) was confirmed to be transferred to the blood (Figure S1). The number of peripheral‐blood Muse cells increased further when the S1PR2 agonist was administered subcutaneously (S1PR2 agonist group), an effect that was counteracted by prior administration of the S1PR2‐specific antagonist JTE‐013 (S1PR2 agonist + antagonist group; Figure 2C), suggesting that the S1PR2 agonist enhanced the mobilisation of Muse cells into the peripheral blood. We previously reported that human Muse cells that predominantly express S1PR2 amongst all the S1PR subtypes preferentially migrate to the post‐infarct border area where the S1P level is highest [13]. We also reported that rabbit‐BM‐Muse cells actively migrate to the S1PR2 agonist SID46371153 in an in vitro study [13]. In the present study, we newly demonstrated in vitro that rabbit‐Muse cells migrated to the serum obtained from a rabbit AMI model (Figure 1A) and an S1PR2 agonist (Figure 1B), but that the migration was suppressed by the S1PR2 antagonist JTE‐013 in a concentration‐dependent manner (Figure 1C). These in vitro studies indicate that the S1PR2 agonist and antagonist efficiently controlled the increase and decrease, respectively, in the number of endogenous peripheral‐blood Muse cells in the rabbit AMI model (Figure 2C).

We also investigated whether the S1PR2 agonist affected the total cell number in the lymphocyte area and monocyte area because it is considered that AMI triggers an early potent activation of myelopoiesis. However, we found no significant difference in the total cell number in the sham, Vehicle and S1PR2 agonist groups under the present conditions. This may be due to the time point when we sampled the peripheral blood after AMI. These results suggest that the S1PR2 agonist did not evoke the mobilisation of immune cells such as lymphocytes and monocytes (Figure 2D,E), but the number of Muse cells increased after AMI and SIPR2 agonist subcutaneous administration. Thus, these findings suggest that the cardiac function recovery, the smaller infarct size, lower number of apoptotic cardiomyocytes, higher vessel concentration and higher marker expression of cardiac and vascular lineages of engrafted Muse cells were due to the increased number of endogenous peripheral‐blood Muse cells and not to an increase in immune cells.

With regard to cardiac function and remodelling, administration of the S1PR2 agonist significantly increased LVEF, LVFS, peak +dP/dt, and peak −dP/dt (Figure 3D–G), and significantly decreased LVDd and LVDs (Figure 3H,I) as compared with the Vehicle group, suggesting the improvement of LV function and attenuation of LV remodelling. The improvement in LV function and attenuation of LV remodelling were almost completely abolished by treatment with the S1PR2 antagonist JTE‐013 (Figure 3D–I). The number of Muse cells in the peripheral blood after AMI positively correlated with LVEF (Figure 3J), suggesting that a higher number of Muse cells in the peripheral blood are associated with improved LV function.

The infarct size was significantly reduced in the S1PR2 agonist group compared with the Vehicle group at 14 days after AMI (Figure 4B,C). This effect was completely abolished by treatment with the S1PR2 antagonist JTE‐013 (Figure 4C). The number of Muse cells in the peripheral blood inversely correlated with the infarct size (Figure 4D), suggesting that a higher number of Muse cells in the peripheral blood is associated with a reduced infarct size. In addition to the attenuation of global LV remodelling such as LV dilation (Figure 3H,I), regional LV remodelling assessed by LV end‐diastolic and LV end‐systolic wall thickness obtained from echocardiography was significantly improved by S1PR2 agonist (Figure S3).

In the present study, we hypothesised that Muse cells in the peripheral blood could be mobilised from the BM by treatment with an S1PR2 agonist. To define whether AMI itself and an S1PR2 agonist mobilise Muse cells from the BM into the peripheral blood and engraft Muse cells to the damaged heart, autologous BM‐Muse cells labelled with GFP were implanted back into the BM of the iliac crest 48 h prior to inducing AMI, and then Vehicle or the S1PR2 agonist SID46371153 was administered subcutaneously at 30 min after AMI. At 2 weeks after AMI, confocal microscopy demonstrated that GFP‐positive Muse cells were detected mainly in the infarct border area (Figure 5A), and the number of GFP‐Muse cells was significantly greater in the S1PR2 agonist group than in the Vehicle group (Figure 5B). Consistent with our previous report [13] and in vitro assessment of the differentiation capacity to cardiac‐lineage cells (Figure 1E–I), the engrafted Muse cells expressed cardiac troponin I and sarcomeric α‐actinin, suggesting that the Muse cells differentiated into cardiomyocytes (Figure 5C–F). The presence of a gap junction between host‐cardiomyocytes and GFP‐Muse cells was suggested by the expression of connexin43 (Figure 5G). The engrafted GFP‐Muse cells expressed CD31, a marker of vascular endothelial cells, in the infarct border area, suggesting differentiation into vascular cells (Figure 5I,J). Furthermore, the numbers of troponin I^+^/GFP^+^, actinin^+^/GFP^+^ and CD31^+^/GFP^+^ cells were significantly greater in the S1PR2 agonist group than in the Vehicle group (Figure 5H,K). The precise number of mobilised Muse cells from the BM of the whole body was not determined due to methodologic limitations. Together, these findings suggest that the infarct size‐reducing effect of the S1PR2 agonist was related to the increase in the number of peripheral‐blood Muse cells.

We previously reported that intravenously administered exogenous Muse cells successfully reduce infarct size by multiple actions, including replacing cardiomyocytes and vessels by differentiation of Muse cells and paracrine effects delivered by Muse cells [13]. The paracrine effects of Muse cells include the upregulation of matrix metalloproteinase 2 and 9, which might suppress scar formation and/or fibrosis [13, 30]; upregulation of hepatocyte growth factor and VEGF, which might lead to neovascularisation and vascular protection [13, 31, 32, 33]; and reduction of apoptotic cardiomyocytes, which might contribute to the reduced infarct size [13]. In the present study, the number of TUNEL^+^ cardiomyocytes was significantly smaller in the S1PR2 agonist group than in the Vehicle group (Figure 4E), and the density of microvessels in the border area was significantly greater in the S1PR2 agonist group than in the Vehicle group (Figure 4F). Therefore, anti‐apoptotic effects and neovascularisation, as well as differentiation of Muse cells into cardiac‐ and vascular‐lineage cells might have contributed to the observed reduction in the infarct size and the improved LV function, similar to findings of a previous study in which exogenous Muse cells were intravenously infused [13]. Besides the improved degree of recovery, the beneficial elements delivered by the increase in endogenous Muse cells enhanced by administration of the S1PR2 agonist did not differ largely from those following infusion of exogenous Muse cells. Thus, the outcome of more efficient mobilisation of endogenous Muse cells appears to be comparable to that of the donor‐derived Muse cell treatment.

Study limitations are that it is unclear whether the protective effects of the S1PR2 agonist, SID46371153, in rabbit myocardial infarction are solely attributable to S1PR2 in Muse cells. This issue may be difficult to resolve without the use of Muse cell‐specific S1PR2 knockout mice. Since it has been reported that sphingosine1‐phosphate receptor 2 has been reported to attenuate reactive oxygen species formation and inhibit cell death [34], the direct effect of the S1PR2 agonist other than Muse cells might have contributed to the beneficial effects in the present study.

The standard and most effective therapy for human AMI is reperfusion of the occluded coronary artery as soon as possible by percutaneous coronary intervention. In AMI patients for whom coronary reperfusion therapy by percutaneous coronary intervention is too late or has failed, cardiac regenerative therapy is required to reconstruct the infarcted cardiac tissue and to prevent LV remodelling and heart failure. Stem cell therapy using exogenous Muse cells is a promising regenerative therapy [13]. As the present study shows, another approach may be to enhance the mobilisation of endogenous Muse cells. Therefore, the present study provides important information for the clinical application of an S1PR2 agonist to enhance the mobilisation of endogenous Muse cells, leading to structural and functional recovery of the infarcted heart.

As clinical implications for treating AMI patients, S1PR2 agonist treatment is a simple, low‐cost method of promoting an increased supply of Muse cells to the peripheral blood compared with the administration of exogenous Muse cells, which requires cell culture and expansion. Some limitations must be overcome, however, because the infarct size‐reducing effect (8.1% reduction) of the S1PR2 agonist treatment was smaller than that produced by exogenous Muse cell treatment (17.6% reduction) [13]. Although many issues still need to be addressed, the development of a clinical grade S1PR2 agonist may provide a new strategy for treating AMI patients.

## Author Contributions

**Shingo Minatoguchi:** formal analysis (equal), investigation (equal), methodology (equal), writing – original draft (equal). **Yoshihisa Yamada:** investigation (equal), methodology (equal). **Noriko Endo:** investigation (equal), methodology (equal). **Hiromitsu Kanamori:** investigation (equal), methodology (equal). **Atsushi Mikami:** investigation (equal), methodology (equal). **Hiroyuki Okura:** supervision (equal). **Shinya Minatoguchi:** formal analysis (equal), funding acquisition (equal), investigation (equal), methodology (equal), project administration (equal), supervision (equal), validation (equal), writing – original draft (equal), writing – review and editing (equal).

## Conflicts of Interest

The authors declare no conflicts of interest.

## Supporting information


Data S1.

## Data Availability

The data that support the findings of this study are available from the corresponding author upon reasonable request.
