# Supplementary material for: Sphingosine‐1‐Phosphate Receptor 2 Agonist Mobilises Endogenous Muse Cells to Repair Damaged Myocardial Tissue in Male Rabbits
Source: J Cell Mol Med. 2025 Apr 17;29(8):e70447. doi: 10.1111/jcmm.70447 (PMC12005397; doi:10.1111/jcmm.70447)
Supplement: Supplementary file 1 — Data S1. [file JCMM-29-e70447-s001.docx]

**Supplemental Online data**

**Materials and Method**

**In vitro Muse cell migration assessment**

Normal serum was collected from blood obtained via the ear artery of the rabbits. For collection of AMI serum, rabbits underwent 30 min of coronary occlusion and 24 h of reperfusion. At 24 h after AMI, serum was collected using the same procedure as described above. Rabbit BM-Muse cells were prepared from BM-mesenchymal stem cells (MSCs) by cell sorting as described previously^1^. A matrigel invasion chamber (Becton Dickinson, Franklin Lakes, NY, USA) was used and the experiment was performed according to the manufacturer’s protocol. Serum (20% vol/vol) from intact or AMI rabbits in α-modified Eagle’s medium (MEM) was placed in the lower chamber under the presence or absence of the S1PR2-specific antagonist (JTE-013; Cayman Chemical, Ann Arbor, MI, USA ; 0.5, 1 and 2 µM) and S1PR2 agonist (SID46371153, Enamine, Ukraine, 1 and 2 μM). In the assays, 3.5×10^4^ BM-Muse cells were placed in the upper chamber and incubated at 37°C, 5% CO_2_ for 24 h. Cells were fixed and stained with the Diff-Quik kit (Sysmex, Kobe, Japan). The number of migrated cells was counted under a light microscope in 5 random fields of each well and the mean of the 5 samples was calculated.

**In vitro differentiation of rabbit peripheral blood-Muse cells into cardiac-lineage cells**

Rabbits were subcutaneously administered 10 mg/kg SID46371153 (S1PR2 agonist). The number of SSEA-3^+^/CD44^+^-rabbit Muse cells in the peripheral blood was significantly increased at 2 h after injecting SID46371153 in intact animals as assessed using a BioRad S3 ^TM^ Cell Sorter (BioRad Laboratories, Inc., Hercules, CA, USA). Therefore, peripheral blood was obtained from the rabbits at 2 h after injecting SID46371153. Approximately 8 mL of peripheral blood was diluted with an equal amount of 0.9% sodium chloride, loaded on a Lymphoprep cushion, and centrifuged at 400x*g* for 20 min at room temperature. The turbid mononuclear cell layer was collected, washed twice with phosphate-buffered saline (PBS), and then plated on collagen type I-coated plastic culture dishes containing alpha-MEM, 10% FBS, and 5 ng/mL basic fibroblast growth factor (bFGF; Reprocell, Yokohama, Japan). Around 40 colony forming units were picked at 10 days and subcultured. Muse cells were collected as SSEA-3^+^ cells (Anti-Stage-Specific Embryonic Antigen-3, clone MC-631, monoclonal antibody [Millipore, Temecula, CA, USA], combined with B-Phycoerythrin Conjugation Kit ab 102871 [Abcam, Tokyo, Japan]) using the BioRad S3 ^TM^ Cell Sorter.

Cardiac-lineage differentiation was analyzed as previously described.^2^ The collected SSEA-3^+^ cells were subjected to suspension culture for 7 days (Stage I). Formed Muse cell clusters in suspension culture were seeded on a collagen type I-coated dish at ~5 clusters/cm^2^ in complete MEM, 10 ng/mL recombinant human bone morphogenetic protein 4 (BMP4; Millipore), 5 ng/mL recombinant human activin A (Millipore), and 5 ng/mL bFGF for 3 days (Stage II); 4 days in complete MEM, 5 ng/mL BMP4, 5 ng/mL activin A, and 0.6 µM dorsomorphin (Wako Pure Chemical, Osaka, Japan, used in place of Noggin as a BMP inhibitor; Stage III). Cells were then reseeded at ~50 cells/cm^2^ on a culture dish coated with 0.25 µg/cm^2^ recombinant laminin fragment (iMatrix-511, Nippi, Tokyo, Japan) in complete MEM containing 1 µM inhibitor of WNT production-3 (Cayman Chemical, used as a Wnt inhibitor in place of Dkk1) and 10 ng/mL vascular endothelial growth factor (VEGF, Wako) for 10 days (Stage IV), then for 7 days with 10 ng/mL VEGF and 5 ng/mL bFGF (Stage V).

**Reverse transcription- quantitative polymerase chain reaction**

Total RNA samples were prepared by extraction using Isogen II (Wako). Rabbit cardiac troponin T and α-actinin mRNAs were quantified using One Step SYBR PrimeScript RT-PCR Kit and Thermal Cycler Dice Real Time System II (Takara Bio, Kusatsu, Japan). The following primers were used for rabbit mRNAs: beta-actin, CATCCGCAAGGACCTGTAC (sense), CCGATCCACACCGAGTACT (antisense); cardiac troponin T, AAGAAGGCGCTGTCCAACAT (sense), AGGTGGTCAATGGCCAGAAC (antisense); sarcomeric α-actinin, GACACCGCCGAGCAAGTCAT (sense), CAGATCACTCTCCCCGTAGA (antisense); GATA4 TCTCGGTCAGCTCCATGTCT (sense), GTGATTATGTCCCCGTGACT (antisense). Beta-actin served as an internal control. The data generated from each reaction were subjected to gene expression analysis using a Thermal Cycler Dice Real Time System (Takara Bio). The amplification program included the initial first strand synthesis at 5 min at 42°C, denaturation step at 95°C for 10 s, followed by 50 cycles of denaturation at 95°C for 30 s, annealing-extension at 60°C for 30 s; and a final extension step at 60°C for 5 min. All data were processed using the ΔΔCT method. Relative expression was calculated defining the most abundant expression as 1 unit.

**Immunocytochemistry**

The primary antibodies used were mouse monoclonal anti-cardiac troponin T (1:200; Thermo Fisher, Waltham, MA, USA ) and rabbit polyclonal anti-sarcomeric α-actinin (1:400; Abcam, Cambridge, UK). The second antibodies were Alexa 488-conjugated goat anti-mouse IgG (1:400, Jackson ImmunoResearch) and Cy3-conjugated goat anti-rabbit IgG (1:400, Jackson ImmunoResearch).

**Rabbit AMI model**

Healthy male Japanese white rabbits (weighing approximately 2.0–2.5 kg) were used, and animals that appeared sick or died during the course of the experiment were excluded from analysis. We used only male rabbits to avoid confounding hormonal effects. The investigators who evaluated the outcomes were blinded to the treatment protocols, and the AMI rabbits were randomly assigned to groups using sealed envelopes for the experiments.

The surgical procedure was performed following to the previously reported methods^.^^1, 3, 4^ Briefly, the rabbits were anesthetized by an intravenous injection of ketamine (10 mg/kg, Ketalar 200mg/20mL, DAIICHI SANKYO COMPANY, LIMITED, Tokyo, JAPAN) and xylazine (3 mg/kg, Selactar 2%, Bayer Yakuhin ltd., Osaka, JAPAN) via ear veins, and additional doses (half dose of each) were given when required throughout the surgery via a jugular vein according to the guidelines of LABIO 21 (<http://www.nichidokyo.or.jp/>) and ARRIVE Guidelines（<https://www.nc3rs.org.uk/arrive-guidelines>）. Once anesthetized, the animals were intubated and ventilated with room air supplemented with a low flow of oxygen using a mechanical ventilator (tidal volume: 25–35 mL, respiratory rate: 20–30/min; Shimano, model SN-480-5, Tokyo, Japan). Serial blood gas analysis was performed, and ventilatory conditions were adjusted to maintain the arterial blood gas within the physiologic range. Surgery was performed under sterile conditions. The carotid artery and jugular veins were cannulated to monitor peripheral arterial pressure and to administer the drugs, respectively. Thereafter, the rabbits were systemically heparinized (500 U/kg), a thoracotomy was performed in the left fourth intercostal space, and the heart was exposed after excising the pericardium. A 4-0 silk suture on a small curved needle was passed through the myocardium beneath the middle segment of the large arterial branch coursing down the middle segment of the anterolateral surface of the left ventricle. Both ends of the silk suture were then passed through a small vinyl tube, and the coronary branch was occluded by pulling the snare, which was fixed by clamping the tube with a mosquito hemostat. Myocardial ischemia was induced for 30 min. Myocardial ischemia was confirmed by ST-segment elevation on electrocardiogram and regional cyanosis of the myocardial surface. Reperfusion was confirmed by myocardial blush over the risk area and a decrease in the ST elevation after releasing the snare. Sham operated animals were prepared using the same surgical procedures as described for the AMI animals except that the arterial branch was not occluded. Animals were sacrificed with an overdose of anesthetics at 2 weeks after AMI.

**Injection of an S1PR2 agonist and antagonist into the AMI rabbit model**

The rabbit AMI model was made as described in the Method section mentioned above. Rabbits received a subcutaneous injection of either 1) 0.5 mL dimethylsulfoxide (DMSO; vehicle group) at 30 min after reperfusion, 2) 10 mg/kg SID46371153 (S1PR2 agonist, Enamine Ltd) dissolved in 0.5 mL DMSO at 30 min after reperfusion (S1PR2 agonist group), or 3) 5 mg/kg JTE-013 (S1PR2-specific antagonist, Cayman Chemical) dissolved in 0.5 ml DMSO immediately after reperfusion, and then 10 mg/kg SID46371153 dissolved in 0.5 mL DMSO at 30 min after reperfusion (S1PR2 agonist+antagonist group). The dose of 10 mg/kg of SID46371153 was determined on the basis of the results obtained in an in vitro study, in which SID46371153 selectively mobilized Muse cells^1^ and based on the previous report in which SID46371153 was used in an in vivo study^5, 6^, and the maximal dose of SID46371153 which could be resolved in DMSO was 10mg/kg. The dose of 5 mg/kg of JTE-013 was also determined on the basis of the results obtained in an in vivo AMI model of rabbits, in which JTE-013 selectively blocked the mobilization of Muse cells ^1^.

**Flow cytometric analysis**

In humans, peripheral-blood Muse cells were isolated as SSEA-3^+^/CD105^+^(mesenchymal marker)^7^. As reported previously, however, CD105 does not work in rabbits and instead CD44 is used as a mesenchymal marker in rabbit.^1, 8^  The number of circulating Muse cells in the peripheral blood was measured as cells double-positive for SSEA-3^+^, a marker of pluripotency, and CD44^+^, a marker of mesenchymal stem cells, at 12 h after AMI. Briefly, a 100-uL aliquot of heparinized whole blood was incubated with anti-SSEA-3 antibody (1:100; Millipore) at 4°C for 30 min followed by staining with secondary antibody fluorescein isothiocyanate-conjugated anti-rat IgM (1:100; Jackson ImmunoResearch, West Grove, PA, USA) and anti-rabbit CD44 (1:10, ANTIGENIX AMERICA Inc., West Hills, NY, USA) in combination with a B-Phycoerythrin Conjugation Kit (ab102871; Abcam) at 4°C for 30 min. Red blood cells were lysed by adding lysing solution (BD Bioscience, San Jose, CA, USA). After washing with PBS, the cells were analyzed using the BioRad S3^TM^ Cell Sorter (488/561 nm) 145-1002 using S3 ProSort Software. For analysis of peripheral-blood Muse cells, single cell analysis software (Flow Jo, LLC, Ashland, OR, USA) was used. The number of whole cells in the lymphocyte and monocyte areas, both of which comprise mononuclear cells, was measured.

**Plasma troponin T measurement**

To confirm that the induction of myocardial infarction was equivalently performed, blood samples from each group were collected from the eart artery 12 h after AMI, and plasma troponin T was measured by electrochemiluminescence immunoassay (LSI Medical Corporation, Tokyo, Japan)

**Physiologic studies**

On day 14 after AMI, the arterial blood pressure and heart rate were measured via a catheter introduced into the carotid artery. Echocardiography (SSD2000, Aloka Co., Ltd., Tokyo, Japan) was performed and the LVEF, LV fractional shortening (LVSF), and LV end-diastolic (LVDd) and end-systolic diameters (LVDs) were obtained by using M-Mode echocardiographic imaging. A micro-manometer-tipped catheter (SPR 407, Millar Instruments, Edwards Lifesciences LLC, Irvine, CA, USA) was inserted into the LV to record peak ±dP/dt and peak -dP/dt. The timing of echocardiography and catheter test was just before the sacrifice of the rabbits 14 days after AMI. The rabbits were anaesthetized by an intravenous injection of ketamine (10 mg/kg, Ketalar 200mg/20mL, DAIICHI SANKYO COMPANY, LIMITED, Tokyo, JAPAN) and xylazine (3 mg/kg, Selactar 2%, Bayer Yakuhin ltd., Osaka, JAPAN) via ear veins,

All measurements were obtained by 2 investigators blinded to the treatment.

**Myocardial infarct size measurement**

Animals were sacrificed with an overdose of pentobarbital (150 mg) at 2 weeks after AMI. The heart was excised at 2 weeks, and the LV was weighed and sectioned into 7 transverse slices parallel to the atrioventricular ring. Each slice was fixed in 10% buffered formalin for 4 h, embedded in paraffin, and cut into 4-μm-thick sections with a microtome. Transverse LV slices at the papillary muscle level were stained with Masson-Trichrome. The LV wall areas, infarct areas, and non-infarct areas were calculated using image analysis software (Win ROOF, version 7.4, Mitani Corporation, Tokyo, Japan) connected to a light microscope (BZ-8000, KEYENCE, Osaka, Japan). The mean of 2 transverse LV slices at the papillary muscle levels was obtained. The myocardial infarct size was calculated as the percent of the Masson-Trichrome-positive area to the LV area. We used Masson Trichrome staining for the measurement of the infarct size as a percentage of LV, because the infarct size scar size as a percentage of LV can also be used at time points after 72 hours of reperfusion^1,3,9^ because of remodeling due to scar shrinkage within the infarct as well as the Evans blue dye-TTC method ^9^. However, the infarct size as a percentage of area at risk obtained in the initial stage of the experiment using Evance blue dye-TTC method^9^ was also shown in the Result section in the Online Supplemental data.　Furthermore, to investigate the infiltration of inflammatory cells into the infarct border area, we used Hematoxylin-Eosin (HE) staining.

Comparisons were made by 2 investigators under blinded conditions. The sections were observed in 20 random high-power fields (x 400) in each sample using a light microscope.

**Immunohistochemistry for CD31 using paraffin sections**

After deparaffinization, 4-μm-thick sections were incubated with a primary antibody against anti-CD31 (1:100; Dako, Santa Clara, CA, USA), followed by horseradish-peroxidase (HRP)-conjugated anti-rabbit IgG and detected by the HRP-3,3'-diaminobenzidine system (Wako Pure Chemical, Osaka, Japan). The anti-CD31 primary antibody (Dako, Santa Clara, CA, USA) was diluted 1:100, incubated overnight at 4 °C and binding developed with the Vectastain ABC kit (200× dilution, PK-4001, Vector Laboratories, Burlingame, CA, USA). The nuclei were counterstained with hematoxylin. The sections were observed in 20 random high-power fields (x 400) by a light microscope.

**Effect of an S1PR2 agonist on cardiomyocyte apoptosis**

Rabbits underwent 30 min of coronary occlusion and 3 days of reperfusion. Then, 0.5 mL DMSO as a vehicle or 10 mg/kg (n=5) SID46371153 (S1PR2 agonist) dissolved in 0.5 mL DMSO (n=5) was subcutaneously administered at 30 min after reperfusion. On day 3 after reperfusion, rabbits were killed under deep anesthesia for removal of the heart, and heart tissue samples were used for the detection of terminal deoxynucleotidyl transferase dUTP nick end labeling (TUNEL)-positive cardiomyocytes. Fixed transverse ventricular slices were embedded in paraffin, and 4-mm-thick sections were deparaffinized by washing in 100% xylene and a descending ethanol series (from 100% ethanol twice to 80% ethanol once and 60% ethanol once). The sections were stained with hematoxylin. DNA fragments were determined using an ApopTag in situ apoptosis detection kit (ApopTag, Oncor Inc, Dallas, TX, USA.). The DNA nick was labeled according to the manufacturer’s instructions. After TUNEL, the sections were counterstained with hematoxylin. Cardiomyocytes in the peri-infarct area were counted under light microscopic analysis. In each specimen, cardiomyocytes with counterstained nuclei were counted in 20 random high-power fields (x 400). Myocytes with a nucleus clearly labeled with diaminobenzidine were defined as TUNEL-positive.

Further to determine TUNEL positive cardiomyocyte, tissue sections stained with Fluorescein-FragEL (green, Oncogene Research Products, Boston, MA, USA) were also labeled with anti-myglobin antibody (1:1000, Dako) followed by Alexa 568 (red, Molecular Probes, Eugene, OR, USA). These sections were then counter stained with Hoechst 33342 (blue) and observed under confocal microscope (C2; Nikon, Tokyo, Japan). The cell showing red cell body and green nucleus was recognized apoptotic cardiomyocyte.

**Transplantation of autologous green fluorescent protein-labeled-Muse cells into the BM followed by administration of an S1PR2 agonist**

Approximately 2 mL of BM aspirate was collected from the left or right iliac crest of each rabbit and mononuclear cells were isolated using Histopaque-1077 (MilliporeSigma, St. Louis, MO, USA). The mononuclear cell layer was collected with a pipette; washed twice; re-suspended in culture medium comprising α-MEM, 10% (vol/vol) FBS, 2.5 ng/mL bFGF, 2 mM GlutaMAX I (GIBCO, Carlsbad, CA, USA), and 0.1 mg/mL kanamycin; and plated onto culture dishes. After 2 days, floating cells were washed off and adherent cells were sub-cultured upon reaching confluency. Fourth generation cells were collected and used as rabbit BM-MSCs. They were then introduced with lentivirus-green fluorescent protein (GFP). GFP-labeled Muse cells were obtained from GFP-labeled MSCs by incubating with an anti-SSEA-3 antibody (1:100; Millipore) and staining with a secondary antibody, DyLight649-conjugated anti-rat IgM (1:100; Jackson ImmunoResearch). SSEA-3+ cells were sorted by a BD FACS AriaTM II cell sorter (Becton Dickinson), as previously described.^10, 11^

Autologous BM-Muse cells labeled with GFP (approximately 3x10^5^ cells) were returned into the BM cavity of the left and right iliac crests in each rabbit according to the previously reported method^3^. This method is confirmed that the implanted mononuclear cells were engrafted into the bone marrow and forming bone marrow cells at 2 weeks after implantation^3^. At 48 h later, rabbits underwent 30 min of coronary occlusion and reperfusion, and then, 0.5 mL DMSO (n=5) or 10 mg /kg SID46371153 (S1PR2 agonist, n=5) was subcutaneously administered at 30 min after coronary reperfusion, and the rabbits were followed up for 2 weeks. The rabbits were then killed by injection of an overdose of anesthesia and fixed with 4% paraformaldehyde in PBS via the coronary artery. Cardiac tissue samples were obtained and snap-frozen in liquid nitrogen for assessment by confocal microscopy (CS-1; Nikon, Tokyo, Japan) to evaluate the differentiation of Muse cells. The sections were observed in 20 random high-power fields (400x) in each sample.

**Immunohistochemistry using cryosections**

Rabbits were killed by an overdose of anesthesia at 2 weeks after AMI and fixed with 4% paraformaldehyde in PBS via the coronary artery. Cryosections (8-μm thick) were cut and incubated with primary antibodies for chicken anti-GFP (1:1000, Abcam), mouse anti-cardiac troponin I (1:50; Millipore), mouse anti-α-actinin (1:100; MilliporeSigma), anti-connexin43 (1:100; Abcam), or anti-CD31 (1:50; Dako). The secondary antibodies used were donkey anti-chicken IgY-Alexa488 (1:500), donkey anti-mouse IgG-Alexa 568 (1:500), or donkey anti-goat IgG-Alexa 568 (1:500). Nuclei were identified by 4',6-diamidino-2-phenylindole staining (1:1000; MilliporeSigma). Images were captured using a confocal laser scanning microscope (CS-1; Nikon).

**Measurement of plasma levels of** **SID46371153 (S1PR2 agonist**)

Blood samples were collected from the ear artery at 12 h after AMI to measure plasma levels of the S1PR2 agonist SID46371153　in the sham, vehicle, S1PR2 agonist, S1PR2 agonist + S1PR2 antagonist (JTE013) groups. Plasma SID46371153 levels were measured by the LC-MS/MS system (API 4000, AB/MDS Sciex, Framingham, MA, USA) at Toray Research Center (Kamakura, Japan).

**Results**

**Plasma levels of SID46371153**

The plasma levels of SID46371153 (S1PR2 agonist) at 12 h after AMI was 2.829 ± 0.265 ng/mL in the S1PR2 agonist group and 2.580 ± 0.269 ng/mL in the S1PR2 agonist + antagonist (JTE-013) group but not detected in the sham or vehicle group (Fig. S1).

**Fig. S1 Plasma levels of SID46371153 (S1PR2 agonist) at 12 h after AMI**


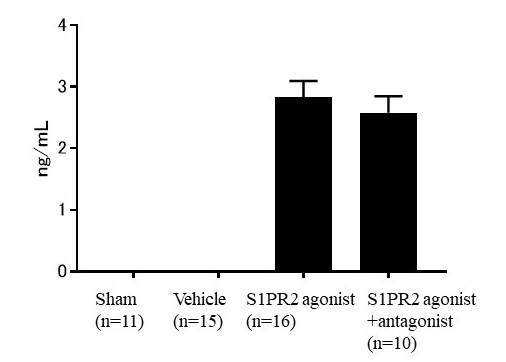


**Fig.S2 Cardiac function of rabbits at baseline before AMI obtained by echocardiography**

LVEF=75.4±1.8 % (n=10), LVFS=41.1±1.6% (n=10), LVDd=14.8±0.7 mm (n=10), LVDs=8.8±0.5 mm (n=10)

**Fig.S3　Echocardiographic LV end-diastolic wall thickness and LV end-systolic wall thickness (*:p<0.05. **:p<0.01, ***:p<0.001)**

In the Vehicle group (n=15), LV end-diastolic wall thickness was 1.53±0.05 mm in the infarct area and 1.69±0.04 mm in the remote area, and LV end-systolic wall thickness was 1.51±0.07 mm in the infarct area and 1.81±0.08 in the remote area.

In the S1PR2 agonist group (n=16), LV end-diastolic wall thickness was 1.80±0.02 mm in the infarct area and 1.89±0.03 mm in the remote area, and LV end-systolic wall thickness was 2.08±0.08 mm in the infarct area and 2.37±0.09 in the remote area. In the Vehicle group, there was no difference between LV end-diastolic wall thickness and LV end-systolic wall thickness both in the infarct and remote areas. In the S1PR2 agonist group, LV end-systolic wall thickness was significantly greater than LV end-diastolic wall thickness both in the infarct area (p<0.05) and remote area(p<0.05).

The ratio of LV end-systolic/LV end-diastolic wall thickness was greater in the S1PR2 agonist group than in the Vehicle group both in the infarct and remote areas.

**Fig.S4 The myocardial infarct size as a percentage of the area at risk obtained by using Evans blue dye-TTC method**

**The infarct size as a percentage of area at risk was significantly smaller in the S1PR2 agonist group (n=8) than in the vehicle group (n=8). ***: p<0.001**

**Fig.S5 The inflammatory cell infiltration into the infarct border area and remote area**

(The sections were observed in 20 random high-power fields (x 200) in each sample using a light microscope.)

Hematoxylin-Eosin staining of the heart tissue showed that the inflammatory cells were observed at the infarct border area both in the vehicle and S1PR2 agonist groups, but the number of inflammatory cells were not different between both groups at 2 weeks after AMI. The inflammatory cells were hardly observed in the remote area both in the vehicle and S1PR2 agonist groups (Fig.S5 in the supplemental online data)

**References**

1. Yamada Y, Wakao S, Kushida Y, Minatoguchi S, Mikami A, Higashi K, Baba S, Shigemoto T, Kuroda Y, Kanamori H, Amin M, Kawasaki M, Nishigaki K, Taoka M, Isobe T, Muramatsu C, Dezawa M, Minatoguchi S. S1P-S1PR2 axis mediates homing of Muse cells into damaged heart for long-lasting tissue repair and functional recovery after acute myocardial infarction. *Circ Res* 2018;**122**:1069-1083.
2. Amin M, Kushida Y, Wakao S, Kitada M, Tatsumi K, Dezawa M. Cardiotrophic growth factor-driven induction of human Muse cells into cardiomyocyte-like phenotype. *Cell Transplant* 2018;**27**:285-298.
3. Minatoguchi S, Takemura G, Chen XH, Wang N, Uno Y, Koda M, Arai M, Misao Y, Lu C, Suzuki K, Goto K, Komada A, Takahashi T, Kosai K, Fujiwara T, Fujiwara H. Acceleration of the healing process and myocardial regeneration may be important as a mechanism of improvement of cardiac function and remodeling by postinfarction granulocyte colony-stimulating factor treatment. *Circulation* 2004;**109**:2572-2580.
4. Chen XH, Minatoguchi S, Kosai K, Yuge K, Takahashi T, Arai M, Wang N, Misao Y, Lu C, Onogi H, Kobayashi H, Yasuda S, Ezaki M, Ushikoshi H, Takemura G, Fujiwara T, Fujiwara H. In vivo hepatocyte growth factor gene transfer reduces myocardial ischemia-reperfusion injury through its multiple actions. *J Card Fail* 2007;**13**:874-883.
5. Park SW, Kim M, Brown KM, D’Agati VD, Lee HT. Inhibition of sphingosine1-phosphate receptor 2 protects against renal ischemia-reperfusion injury. J Am Soc Nephrol 2012; **23**: 266-280
6. Yan H, Yi S, Zhuang H, Wu L, Wang DW, Jiang J. Sphingosine-1-phosphate ameliorates the cardiac hypertrophic response through inhibiting the activity of histone deacetylase-2. Int J Mol Med 2010; **42**: 572-577
7. Tanaka T, Nishigaki K, Minatoguchi S, Nawa T, Yamada Y, Kanamori H, Mikami A, Ushikoshi H, Kawasaki M, Dezawa M, Minatoguchi S. Mobilized Muse cells after acute myocardial infarction predict cardiac function and remodeling in the chronic phase. *Circ J 2018;* **82**:561-571.
8. Vasicek J, Kovac M, Balazi A, Kulikova B, Tomkova M, Olexikova L, Curlej J, Bauer M, Schnabl S, Hilgarth M, Hubmann R, Shehata M, Makarevich AV, Chrenek P. Combined approach for characterization and quality assessment of rabbit bone marrow-derived mesenchymal stem cells intended for gene banking. *N Biotechnol* 2020;**54**:1-12.
9. Yamada Y, Kobayashi H, Iwasa M, Sumi S, Ushikoshi H, .Aoyama T, Nishigaki K, Takemura G, Fujiwara T, Fujiwara H, Kiso M, Minatoguchi S. Post-infarct active cardiac-targeted delivery of erythropoietin by liposomes with sialyl Lewis X repairs infarcted myocardium in rabbits. Am J Physiol Heart Physiol 2013; **304**: H1124-H1133
10. Kuroda Y, Kitada M, Wakao S, Nishikawa K, Tanimura Y, Makinoshima H, Goda M, Akashi H, Inutsuka A, Niwa A, Shigemoto T, Nabeshima Y, Nakahata T, Nabeshima Y, Fujiyoshi Y, Dezawa M. Unique multipotent cells in adult human mesenchymal cell populations. *Proc Natl Acad Sci U S A* 2010;**107**:8639-8643.
11. Wakao S, Kitada M, Kuroda Y, Shigemoto T, Matsuse D, Akashi H, Tanimura Y, Tsuchiyama K, Kikuchi T, Goda M, Nakahata T, Fujiyoshi Y, Dezawa M. Multilineage-differentiating stress-enduring (Muse) cells are a primary source of induced pluripotent stem cells in human fibroblasts. *Proc Natl Acad Sci U S A* 2011;**108**:9875-9880.
